# Lyme arthritis outcomes in children: a single center cohort study

**DOI:** 10.1186/1546-0096-10-S1-A38

**Published:** 2012-07-13

**Authors:** Brandt P Groh, Nicholas J Ahn

**Affiliations:** 1 Hershey Medical Center, Hershey, PA, USA

## Purpose

To identify potential risk factors for chronic Lyme arthritis in children and to describe the course of chronic arthritis in our cohort.

## Methods

Children evaluated in the pediatric rheumatology clinics of Penn State Children’s Hospital and diagnosed with Lyme arthritis between 2002 and 2010 were reviewed from initial contact to the most recent follow-up documentation available. The diagnosis of Lyme arthritis in all cases was based on the presentation of synovitis and/or enthesitis in combination with positive Western blot assays by CDC criteria. Children with transient arthritis (< 3 mo duration) were compared with children manifesting chronic arthritis (>3 mo) by presenting laboratory features and clinical features throughout their follow-up.

## Results

127 study subjects were included in this review after excluding 19 lost to follow-up and 6 with insufficient data for review. The median age of this cohort was 8.5 yrs (range 1.3 – 17.9). 61% were male and 39% female. 83% were Caucasian. Median follow-up was 13.5 months (range 3 – 49) for the chronic subgroup versus 2.6 months for the transient subgroup. Significant differences by bivariate analysis are listed in table 1.

**Table 1:** 

Chronic arthritis	Transient (n = 92)	Chronic (n = 35)	P value
Median age	8.5 yrs (1.3-17.5)	11.2 yrs (2.5-17.9)	=0.016†
Additional joints	15%	71%	<0.001‡
>3 symptomatic joints	19%	46%	=0.004‡
1^st^ – 2^nd^ degree relative with rheumatic dz	26 (24%)	41%	=0.023‡
Spondyloarthropathy disease features	0%	49%	<0.0001‡

† = significance by Wilcoxon rank-sum test. ‡ = significance by Chi-square test.

Only the involvement of new joints beyond the initial presentation and spondyloarthropathy features remained significant predictors of chronicity by stepwise logistic regression analysis. There were no significant differences between subgroups in time to initial treatment, presence of early dissemination symptoms, ANA positivity, or initial ESR values. Rocephin was prescribed in 28% of the chronic subgroup versus 9% of the transient subgroup. Only 14/35 patients with chronic arthritis resolved their inflammatory joint findings within the period of follow-up, leaving 17% of the entire cohort with long term chronic arthritis. Median time to resolution of chronic arthritis was 11 months (range 4 – 66) in these 14 patients.

## Conclusion

Similar to previous cohort studies, we found older age at presentation to be associated with persistence of arthritis. Our study is unique in identifying rheumatic disease in 1^st^ and 2^nd^ degree relatives as a chronicity risk factor. In fact, 18/35 (51%) of the children in our chronic arthritis subgroup were reclassified as probable or definite rheumatic diseases: 9 spondyloarthropathy, 6 psoriatic arthritis, 2 JIA, and 1 isolated TMJ synovitis. Early spondyloarthropathy disease features (in particular enthesitis and inflammatory back pain) were highly correlated with a chronic arthritis course in this cohort. Our cure rate of 83% is lower than that reported in previous studies. Around 25% of children with Lyme arthritis will experience chronic musculoskeletal inflammation following appropriate antibiotic therapy. A significant proportion of these children may have a rheumatic predisposition, as evidenced by family histories and subsequent rheumatologic diagnoses.

## Disclosure

Brandt P. Groh: None; Nicholas J. Ahn: None.

